# Targeting the RHOA pathway improves learning and memory in adult *Kctd13* and 16p11.2 deletion mouse models

**DOI:** 10.1186/s13229-020-00405-7

**Published:** 2021-01-13

**Authors:** Sandra Martin Lorenzo, Valérie Nalesso, Claire Chevalier, Marie-Christine Birling, Yann Herault

**Affiliations:** 1 Université de Strasbourg, CNRS, INSERM, Institut de Génétique Biologie Moléculaire et Cellulaire - UMR 7104 - U1258, IGBMC, 1 rue Laurent Fries, 67404 Illkirch Cedex, France; 2 Université de Strasbourg, CNRS, INSERM, CELPHEDIA-PHENOMIN-ICS, Institut Clinique de La Souris, 1 rue Laurent Fries, 67404 Illkirch Cedex, France

**Keywords:** Copy number variation, Neurodevelopment, Intellectual disability, Autism spectrum disorders, KCTD13, Mouse model, Recognition memory, Preclinical treatment

## Abstract

**Background:**

Gene copy number variants play an important role in the occurrence of neurodevelopmental disorders. Particularly, the deletion of the 16p11.2 locus is associated with autism spectrum disorder, intellectual disability, and several other features. Earlier studies highlighted the implication of *Kctd13* genetic imbalance in 16p11.2 deletion through the regulation of the RHOA pathway.

**Methods:**

Here, we generated a new mouse model with a small deletion of two key exons in *Kctd13.* Then, we targeted the RHOA pathway to rescue the cognitive phenotypes of the Kctd13 and 16p11.2 deletion mouse models in a pure genetic background. We used a chronic administration of fasudil (HA1077), an inhibitor of the Rho-associated protein kinase, for six weeks in mouse models carrying a heterozygous inactivation of *Kctd13*, or the deletion of the entire 16p11.2 BP4-BP5 homologous region.

**Results:**

We found that the small *Kctd13* heterozygous deletion induced a cognitive phenotype similar to the whole deletion of the 16p11.2 homologous region, in the Del/+ mice. We then showed that chronic fasudil treatment can restore object recognition memory in adult heterozygous mutant mice for *Kctd13* and for 16p11.2 deletion. In addition, learning and memory improvement occurred in parallel to change in the RHOA pathway.

**Limitations:**

The *Kcdt13* mutant line does not recapitulate all the phenotypes found in the 16p11.2 Del/+ model. In particular, the locomotor activity was not altered at 12 and 18 weeks of age and the object location memory was not defective in 18-week old mutants. Similarly, the increase in locomotor activity was not modified by the treatment in the 16p11.2 Del/+ mouse model, suggesting that other loci were involved in such defects. Rescue was observed only after four weeks of treatment but no long-term experiment has been carried out so far. Finally, we did not check the social behaviour, which requires working in another hybrid genetic background.

**Conclusion:**

These findings confirm KCTD13 as one target gene causing cognitive deficits in 16p11.2 deletion patients, and the relevance of the RHOA pathway as a therapeutic path for 16p11.2 deletion. In addition, they reinforce the contribution of other gene(s) involved in cognitive defects found in the 16p11.2 models in older mice.

## Introduction

Genetic copy number variants of the 16p11.2 locus are an important risk factor for multiple neurodevelopmental disorders [1–5]. The most recurrent 16p11.2 rearrangements, deletion and reciprocal duplication, induce intellectual disability [6] and Autism Spectrum Disorders [7–12]. In addition, they are associated with other neuropsychiatric disorders, such as epilepsy [11], attention deficit/hyperactivity disorder [13], schizophrenia and bipolar trouble [14]. Body mass index phenotypes and abnormal head size have also been reported in these 16p11.2 rearrangements [11–13, 15, 16]. The most frequent variation of the 16p11.2 region corresponds to change in the genetic interval between *SULT1A1* *and* *SPN1*, encompassing 600 kb and 32 genes. Nevertheless, a study conducted in 2011 found a microdeletion of the 118 kb region, containing MVP, SEZ6L2, CDIPT, ASPHD1 and KCTD13 inside the 16p11.2 genetic interval, linked to Autism Spectrum Disorder in a family over three generations [17]. Thus, this *MVP-KCTD13* subregion should play a potential key role in the neuropsychiatric features linked to 16p11.2 rearrangement.

Modelling 16p11.2 rearrangements using animal models recapitulates human genetic data. Indeed, three mouse models were developed carrying deletions of the 16p11.2 homologous genetic interval [18–20] of slightly different size. Nevertheless, they were found to share common phenotypes including hyperactivity, repetitive behaviours, and deficits in object memory [18–20] that are related to certain human cognitive and behavioural features found in the 16p11.2 deletion syndrome [8].

Several studies have been carried out to find the specific brain regions, developmental periods, networks and pathways impacted by the 16p11.2 deletion. In particular, the development of dynamic spatio-temporal networks of 16p11.2 genes integrating data from the brain developmental transcriptome with data from physical interactions of 16p11.2 proteins supported the role of KCTD13 as a protein that complexes with CULLIN 3 (CUL3) ubiquitin ligase, regulating the protein levels of the Ras homolog family member A (RHOA) [21]. The known important functions of the Rho GTPase signalling pathway in brain morphogenesis at early stages of brain development led to the assumption that *KCTD13* dosage changes in 16p11.2 deletion or duplication carriers influence RHOA levels and results in impaired brain morphogenesis and cell migration during the foetal stages of brain development [21]. This hypothesis was supported by later studies showing the implication of KCTD13 in abnormal brain size in downregulation experiments in zebrafish [22].

In addition, the *Kctd13*^+/−^mouse model showed a reduction of the number of functional synapses, with a decrease of dendritic length, complexity and dendritic spine density due to increased levels of RHOA [23]. These alterations were reversed by RHOA inhibition with rhosin, strengthening the potential role of RHOA as a therapeutic target. Interestingly, the recognition deficit was not seen with this model in another more complex paradigm in which the recognition test was based on 3 objects [23], a more challenging task with multiple recognition steps, involving slightly different memory mechanisms. Also, recent studies revealed dendritic spine maturation alterations of hippocampal pyramidal neurons in another *Kctd13*^+/−^ mouse model [24]. This model also presented a deficit in recognition and location memory in a paradigm with two objects similar to the phenotype detected in previous studies done on three distinct 16p11.2 *Del/+* mouse models [18–20]. Surprisingly, no change in RHOA protein level was detected in the most recent study [24].

Despite these investigations, how KCTD13 levels regulate the RHOA signalling pathway, and its implication in the phenotypes associated with the 16p11.2 syndromes, remain unclear. Thus, we decided to explore the role of *Kctd13* by engineering a new loss-of-function allele, removing only exons 3 and 4 with CrispR/Cas9, instead of the whole gene [23] or exon 2 [24]. Then, we characterized the novel heterozygous mouse model and compared its outcome with the deletion of 16p11.2 in a selected number of tests.

Furthermore, the integration of our results with earlier studies led us to hypothesize that the 16p11.2 deletion leads to an over-activation of the KCTD13-CUL3-dependent RHOA pathway. Consequently, if we assume that RHOA/ROCK pathway over-activation causes behavioural and learning alterations in the 16p11.2 deletion, inhibiting this pathway should improve the *Kctd13*^+/−^ and the 16p11.2 *Del/+*-associated phenotypes. We decided therefore to treat our mice with fasudil (HA1077), an inhibitor of the ROCK kinase from the RHOA pathway. We used a similar strategy to counterbalance the behavioural impairments of the Oligophrenin-1 mouse model of intellectual disability [25]. Thus, we initially planned to characterize the behaviour phenotypes of the new *Kctd13* haplo-insufficient model and compare the outcome with the 16p11.2 *Del/+* mice. Then, we set up a chronic administration of fasudil (HA1077) in adult *Kctd13*^+/−^ and 16p11.2 *Del/+* mice (this work and [20]) and evaluated the behaviour of the treated versus the non-treated animals. Next, we carried out a molecular analysis of key members of the RHOA pathway.

## Materials and methods

### Mouse lines, genotyping and ethical statement

Two mouse models were used in the study. The 16p11.2 mouse model corresponds to the Del(7*Sult1a1-Spn*)6Yah mouse model [20], noted *Del/+* here. The line was kept on a pure C57BL/6N inbred genetic background. The deletion allele was identified by PCR using primers Fwd1 (5′-CCTGTGTGTATTCTCAGCCTCAGGATG-3′) and Rev2 (5′-GGACACACAGGAGAGCTATCCAGGTC-3′) to detect a specific band of 500 bp while the wild-type allele was identified using Fwd1 and Rev1 (5′-GGACACACAGGAGAGCTATCCAGGTC-3′) primers to detect the presence of a 330 bp fragment. The PCR program was: 95 °C/5 min; 35 × (95 °C/30 s, 65 °C/30 s, 70 °C/1 min), 70 °C/5 min.

The *Kctd13*^*em2(IMPC)Ics*^ knock-out mouse was generated by the CRISPR/Cas9 technology [27] in the C567BL/6N genetic background (Additional file 1: Supplementary Fig. 1A). Two pairs of sgRNAs, one pair located upstream and the other pair downstream of the target region, were selected to delete exons 3 and 4 of the gene. Both pairs of sgRNAs (showing a cut) and Cas9 mRNA were microinjected into fertilized eggs derived from super-ovulated sexually immature C57BL/6N female mice (4–5 weeks olds). Injected embryos cultured in vitro were implanted in the oviducts of pseudo-pregnant females. The deletion of *Kctd13* in *Kctd13*^*em2(IMPC)Ics*^ (noted here *Kctd13*^+/−^) was confirmed by PCR using primers Ef (5′-ACCTCTTAGCTGGGCATGCTAAATT-3′) and Xr (5′-AGCCTATGCTAACTATTATCACAGG-3′) and the sequence of the deleted fragment. The PCR reaction gave deletion and wild-type products of 429 and 668 bp long, respectively (Additional file 1: Supplementary Fig. 1B). The PCR program was: 94 °C/5 min, 35 X (94 °C/30 s; 60 °C/30 s; 72 °C/30 s), 72 °C/5 min. This set of primers was also used for genotyping (Additional file 1: Fig. 1B). The model was validated by detecting the decreased level of KCTD13 in the mutant hippocampi compared to control (Additional file 1: Supplementary Fig. 1C). All the mouse models are available through the Infrafrontier European repository or the International Mouse Phenotyping consortium.

### Chronic fasudil treatment

**Fig. 1:**
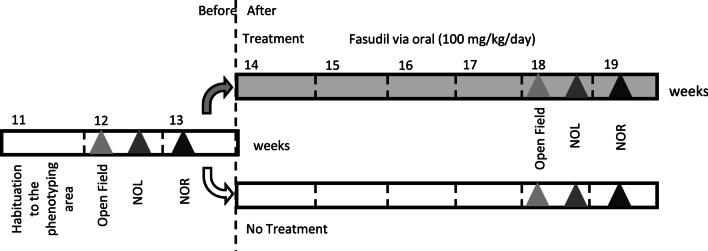
Outline of the behavioral pipeline used for investigating the therapeutic effects of the drug fasudil in the *Kctd13*^+/−^ and 16p11.2 *Del/+* mouse models. We used 3 independent subcohorts of animals for each mouse line. 12-week-old mice were subjected to different behavior and learning tests, after prior habituation to the phenotyping zone. At 14 weeks, each cohort was divided into two groups. The first group started the fasudil treatment and the second group followed the cognitive characterization without treatment. *NOL* novel object location, *NOR* novel object recognition

In this study, we developed a protocol for a pre-clinical treatment (Fig. 1) with the drug fasudil hydrochloride or HA1077 (F4660, LC laboratories Boston, MA, USA). At weaning, control wild type littermates and heterozygous male mice, either *Kctd13*^+/−^or *Del/+* , were taken from several litters and housed in groups of 4–2 individuals in ventilated cages (Green Line, Techniplast, Italy), where they had free access to water and diet (D04 chow diet, Safe, Augy, France). Animal bedding (Poplar litter, AB 3 autoclavable, AniBed, Pontvallain, France) was changed once a week. At 11 weeks, animals were transferred from the animal facility to the phenotyping area. The temperature was kept at 21 ± 2 °C, and the light cycle was controlled as 12 h light and 12 h dark (lights on at 7 am).

At 12 weeks of age, three independent cohorts of mice for each line, with wild-type (wt) and mutant littermates, were subjected to a battery of behavioural tests (see below) for 2 weeks. Then, 14-week-old mice were randomly divided into 2 groups: one treated with fasudil administrated orally ad libitum in drinking water to reach a dose of 100 mg/kg/day and a second with no treatment. The dose was estimated at 152.7 mg in a feeding bottle (250 ml) changed twice a week, taking into account the previous study in which we verified that the drinking volume was about 4.6 ml/day/mouse [25]. Four weeks after the beginning of the treatment, 18-week-old mice were challenged once again with the same battery of behavioural tests (see below) and kept under the same treatment condition (Fig. 1). The experiments were conducted blindly for genotype as recommended by the ARRIVE guidelines [28, 29]. All animals injured by their cage companions were excluded from the behavioural tests at the time when they were seen. A second batch of three independent cohorts were processed similarly but without behaviour tests for the molecular analysis of the hippocampal region from treated and non-treated mice. In this case, treated animals started the fasudil treatment at the age of 12 weeks, for 6 weeks. Samples were quickly harvested from 18-week-old mice after euthanasia by cervical dislocation and snap frozen for molecular analyses.

### Behavioural analysis

We used three tests that previously unravelled robust phenotypes in the three 16p11.2 mouse models [18–20]: the open field for the exploration activity, novel object location and novel object recognition for learning and memory in mice. The novel object location (NOL) memory task stimulates the parahippocampal cortex, the entorhinal cortex and the hippocampus [26] whereas the novel object recognition (NOR) memory task is based on the innate preference of rodents to explore novelty involving the perirhinal and entorhinal cortex and the hippocampus.

For the open field (OF), mice were tested in an automated arena (44.3 × 44.3 × 16.8 cm) made of PVC with transparent walls and a black floor, and covered with translucent PVC (Panlab, Barcelona, Spain). The arena was divided into central and peripheral regions (8 cm peripheral zone and 28 cm central zone) and homogeneously illuminated at 150 lx. Each mouse was placed on the periphery of the open field and allowed to explore the apparatus freely for 30 min while 16 × 16 infrared captors (located at the periphery at two different levels) recorded the horizontal and vertical position of the mouse. During each session we measured the total distance travelled, evaluated the habituation of the animal over time, by splitting the data in 10-min intervals, and assessed vertical activity through the number of rears.

The NOL memory task was carried out in the same open field arena as previously described. On the first day, mice were habituated to the arena for 30 min at 150 lx. On the following day, animals went through an acquisition trial during the first 10 min in which they were presented individually to 2 identical objects A. Each object was placed 10 cm away from each of the corners on the north side of the box. The exploration time of objects A (when the animal’s snout was directed towards the object at a distance ≤ 1 cm) was manually recorded with a chronometer. Minimum exploration time was set to 3 s, and mice that did not reach this criterion or show any interest for one object were excluded from the study. A 10-min retention trial (second trial) was conducted 5 min later, when one of the familiar objects was displaced to a novel location (B) on the south side and the exploration time (t) of the two objects was recorded for 10 min. The selection of the new object (right or left) was counter balanced across animals. We used two identical cylindrical objects of black colour with a white circle on top. In this session, the minimum exploration time was also set to 3 s, and mice that did not reach this criterion or show any interest (0 s of exploration) in one object were excluded from the study. We verified that no preference was seen during the exploration of the left and right object. The recognition index (RI) was defined as [*t*B/(*t*A + *t*B) × 100]. An RI of 50% corresponds to the chance level and a significantly higher RI reflects good recognition of which object was moved in between the two sessions.

The NOR test was performed in a circular open field of white PVC with opaque walls and floor 30 cm high and 50 cm diameter. On the first and second days, each mouse was habituated to the arena for 15 min at 60 lx. The following day, we started the NOR sessions. First, each animal was individually subjected to a 10 min acquisition trial for the presentation of two identical objects A (either marble or dice) placed at the northeast or northwest of the open field arena. The exploration time of both objects A was recorded. 3 h later (retention delay in home cages), a 10 min retention trial (second trial) was performed. One of the identical objects A was replaced with a novel object B at the same position. The new object location was randomly determined for each animal and overall balance assessed. The exploration time of the two objects (familiar object and novel object) was recorded using the NOLDUS video tracking software. The recognition index (RI) was defined as (*t*B/(*t*A + *t*B) × 100). An RI of 50% corresponds to a chance level and a significantly higher RI reflects good recognition memory. Any mice that did not explore the objects for more than 3 s during the acquisition trial or the retention trial or show any interest for one object were excluded from the analysis.

### Western blot

Fresh hippocampal tissues were isolated by rapid decapitation/dissection of naive mice and snap frozen. Then, they were lysed in ice-cold sonication buffer supplemented with Complete™ Protease Inhibitor Cocktail (Roche). Individual samples were disaggregated, centrifuged at 4 °C for 30 min at 14,000 rpm, diluted in 4X Laemmli sample buffer containing β-mercaptoethanol (Bio-Rad, France), and incubated at 95 °C for 5 min. Protein concentration was determined by the Pierce™ BCA Protein Assay Kit (23,225, Thermo Fisher Scientific, Strasbourg). The samples were diluted with sample buffer such that 30 µg of protein were loaded per lane onto 15% polyacrylamide gel. Gels were run and then transferred to nitrocellulose membranes by the Trans-Blot^®^ Turbo™ Transfer System (BioRad, France) using the MIXED MW Bio-Rad Preprogrammed Protocol. Then they were blocked in 5% BSA, 1 X Tris-buffered saline, 0.1% Tween 20 (TBS-T) and incubated with primary antibody for 10 min. Membranes were washed in TBS-T followed by a 10-min secondary antibody incubation using an HRP conjugated Goat anti-Rabbit IgG (A16096, Invitrogen, France) at 2:10,000 using the SNAP i.d.^®^ 2.0 Protein Detection System (C73105, Merck). This apparatus has a vacuum-driven technology and a built-in flow distributor that actively drives reagents through the membrane.

Total levels of KCTD13, RHOA protein and Myosin Light Chain and its phosphorylation product were analysed using Western Blot. Proteins were visualized with Amersham™ Imager 600. Signals were quantified using ImageJ and analysed using Microsoft Excel and GraphPad Prism. We used the following primary antibodies: KCDT13 (dilution 1:250, HPA043524, Atlas Antibodies, Bromma, Sweden), RHOA (dilution 1:1000; cat #2117, Cell Signaling, USA), MLC (dilution 1:1000;cat # 8505, Cell Signaling Technology Europe, B.V., Leiden, The Netherlands) and pMLC (dilution 1:1000; Thr18/Ser19 cat #3674, Cell signalling, Boston, MA, USA). The ratio of protein (or phosphorylated protein) level was estimated against control β-actin protein level [detected with a mouse monoclonal Anti-β-Actin— Peroxidase antibody (A3854 Sigma)] and normalized to untreated wt sample mean.

### Statistical analysis

The statistical analysis was carried out using standard statistical procedures available on the SigmaPlot software (Systat software, San Jose, USA). All outliers were identified using the Grubbs' test from the GraphPad calculator (GraphPad Software, San Diego) or ROUT method with a Q value of 1% from GraphPad Prism 7.01 (GraphPad Software, San Diego) for data with nonlinear regression. We estimated a priori the power of the different variables, scored during the behavioural analysis, to test our H0 hypothesis, taking into account our previous analysis of the Del/+ model [20]. A posteriori we verified that the sample size was sufficient to have a statistical power of 100% for the object location and recognition memory at both ages (Additional file 1: Table 1). Nevertheless the powers of the open field variable “distance travelled” were lower at 12 compared to 18 weeks, certainly due to a change in variability. Based on the power calculation we did not consider the results of the rearing activity from the open field. Data from the behavioural characterization of *Kctd13*^+/−^ and 16p11.2 *Del/+* mouse models were analysed by the Student *t* test (see Additional file 1: Tables 2 and 3 for a summary). One sample *t* test was used to compare recognition index values to the set chance level (50%). Data from post-treatment behavioural phenotyping of both genetic lines were analysed using one-way or two-way ANOVA followed by Tukey’s post-hoc test whenever data presented normal distribution and equal variance. Otherwise, we used the non-parametric Kruskal–Wallis one-way analysis of variance and the Mann–Whitney *U* test. One sample *t* test was also used to compare recognition index values to the set chance level (50%). Western blot data were analysed using a Kruskal–Wallis one-way analysis of variance test between groups followed by a Mann–Whitney *U* test or Student *t* test depending on data distribution. Data are represented as the mean ± SEM and the significant threshold was *p* < 0.05.

## Results

### Phenotypic characterization of the new ***Kctd13***^+/−^ mouse model and comparison with the 16p11.2 ***Del/+*** mouse model

**Fig. 2:**
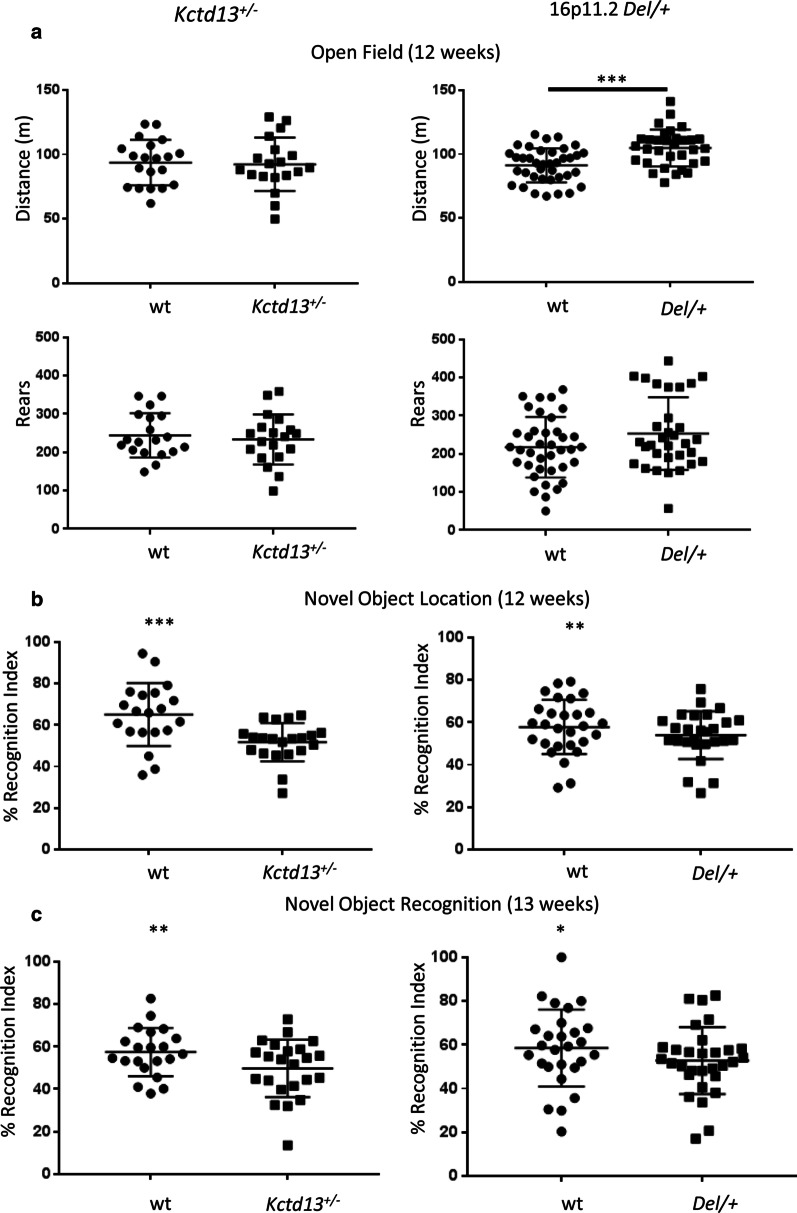
Exploration activity in the **a** open field test, **b** novel object location and **c** novel object recognition of the *Kctd13*^*+/−*^ and the 16p11.2 *Del/+* mouse models at 12 and 13 weeks of age. **a** Male mice from the *Kctd13*^+/−^ line [wt (*n* = 20) and *Kctd13*^+/−^ (*n* = 19)] and from the 16p11.2 *Del/+* line [wt (*n* = 38) and *Del/+* (*n* = 32)] were free to explore the open field for 30 min as a new environment. First, the exploration activity was analysed from the total distance travelled during the test. Next, the adaptation of the mice to the environment was evaluated by dividing the test into periods of 10 min. The *Kctd13*^+/−^ animals showed no alteration compared to their wt littermate whereas the 16p11.2 Del/+ mice showed increased exploratory activity in the distance travelled compared to wt. The *Del/+* mutant individuals displayed a decrease in the area of exploration during the test and thus normal habituation to the new environment. **b** In the NOL test, the recognition index reflects the ability of mice from the two lines [wt (*n* = 20) and *Kctd13*^+/−^ (*n* = 21) littermates; and 16p11.2 *Del/+* (wt (*n* = 28) and *Del/+* (*n* = 27) littermates] to distinguish the new located object from the familiar one after a 5 min retention delay. The *Kctd13*^+/−^ and the *Del/+* male mice showed a deficit in object location recognition memory compared to their wt littermates. **c** In the NOR test, the animals [wt (*n* = 24) and *Kctd13*^+/−^ (*n* = 23) littermates; and wt (*n* = 27) and *Del/+* (*n* = 30) littermates] were challenged to recognize the new object from the familiar object after a 3 h delay. *Kctd13*^+/−^ and the *Del/+* mice showed a poor object recognition memory compared to their respective wt littermates; (*p < 0.05; **p < 0.01; ***p < 0.001)

We created a new *Kctd13* KO mouse model with the deletion of exons 3 and 4, leading to a frameshift at the transition of exons 2 and 5 (Additional file 1: Supplementary Fig. 1A–B). We validated the new allele by DNA analysis for the deletion and the decreased expression of KCDT13 in mutant hippocampi (Additional file 1: Supplementary Fig. 1C), and by phenotyping at the IMPC (www.mousephenotype.org). Then, we evaluated its learning and memory behaviour at 12 weeks of age in the open field, NOL and NOR, and compared the results obtained with the characterization of the 16p11.2 *Del/+* model (Fig. 2). First, we did not detect significant differences in the open field test (12 weeks), when we measured the total distance travelled by *Kctd13*^*+/−*^ mice compared to wt littermates [Fig. 2a left panel, wt (*n* = 20) and *Kctd13*^*+/−*^ (*n* = 19)]. This result was different from the phenotype of the *Del/+* mice [Fig. 2a right panel, wt (*n* = 38) and *Del/+* (*n* = 32)]. Indeed *Del/+* mutant mice were more active with a significant increase in the distance travelled compared to their wt littermates [Student *t* test: total distance: wt vs. *Del/+* *t*_(68)_ = − 4.096; *p* < 0.001], as previously observed [20]. Then, we analysed the distance travelled in 5-min intervals, to observe the habituation to a new environment during the test. We found that the *Kctd13*^*+/−*^ mice experienced a similar habituation to the control individuals (Additional file 1: Supplementary Fig. 2A). Here too, a significant difference was seen in the 16p11.2 *Del/+* carrier mice [paired *t* test: *T*_0–10_ vs. *T*_20–30_ *t*_(31)_ = 14,319; *p* < 0.001]. Although they were more active, the mutant mice showed normal habituation compared to their wild-type littermates.

Afterwards, we analysed the object location memory (Fig. 2b). For this test, animals (wt (*n* = 20) and *Kctd13*^+/−^ (*n* = 21) littermates; and 16p11.2 *Del/+* [wt (*n* = 28) and *Del/+* (*n* = 27) littermates] were scored for their ability to distinguish between a previously presented object that remained in the same location and another object whose position was changed, after a retention delay of 5 min. The recognition indexes for the novel object location showed that, contrary to their wt littermates, both *Kctd13*^+/−^ and 16p11.2 *Del/+* mice were not able to differentiate between the novel and familiar location [one sample *t* test for the *Kctd13*^+/−^ line: wt (*t*_(19)_ = 4.4607; *p* = 0.0003), *Kctd13*^+/−^ (*t*_(16)_ = 0.9170; *p* = 0.3728); and the 16p11.2 *Del/+* model: wt (*t*_(27)_ = 3.2299; *p* = 0.0032), *Del/+* (*t*_(26)_ = 1.8372; *p* = 0.0776)].

Finally, we investigated whether *Kctd13*^+/−^ mice [wt (*n* = 24) and *Kctd13*^+/−^ (*n* = 23) littermates] could discriminate a novel object from a previously explored set of two objects after a retention delay of 3 h in the NOR task and we verified the deficiency found in recognition memory in the 16p11.2 mutant mice (*n* = 30) compared to wt littermates (*n* = 27) (Fig. 2c). Whereas wt animals were able to differentiate objects showing a novel object preference, *Kctd13*^+/−^ mice were not able to discriminate the novel from the familiar object. The deficit was like that seen in the 16p11.2 Del/+ mice [one sample *t* test for the *Kctd13*^+/−^ model: wt (*t*_(23)_ = 3.0558; *p* = 0.0056), *Kctd13*^+/−^ (*t*_(22)_ = 0.0805; *p* = 0.9366); and the 16p11.2 Del/+ model: wt (*t*_(26)_ = 2.5312; *p* = 0.0178), *Del/+* (*t*_(29)_ = 1.0119; *p* = 0.3199)]. Overall, our behavioural analysis showed that the *Kctd13* haploinsufficiency phenocopied the object location and recognition memory deficits seen in the 16p11.2 deletion model in the same pure C57BL/6N genetic background. However, the increased exploration activity found in the 16p11.2 *Del/+* mice was not seen in the *Kctd13*^+/−^ mutant mice.

### Fasudil treatment partially reverses the cognitive impairment in the ***Kctd13***^+/−^ and the 16p11.2 ***Del/+*** mouse models.

**Fig. 3:**
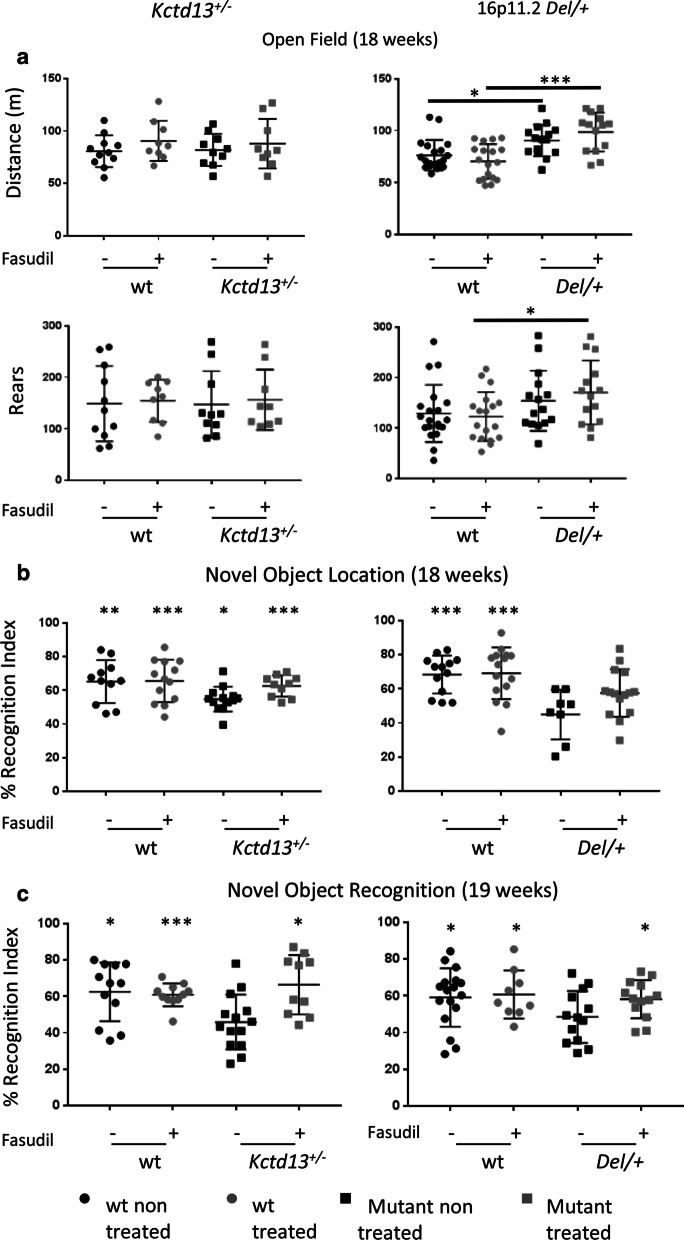
Exploration activity in the **a** open field test, **b** novel object location and **c** novel object recognition of the *Kctd13*^+/−^ and the 16p11.2 *Del/+* **b** mouse models with or without fasudil treatment. **a** The *Kctd13*^+/−^ animals showed no alteration compared to their wt littermate whereas the *Del/+* mice showed increase exploratory activity in the distance travelled during the exploration of the open field for 30 min, which was not affected by the fasudil treatment. **b** For NOL test, recognition index reflects the ability of mice to distinguish the new location of an object from the familiar one after a 5 min retention delay. We observed again that the 16p11.2 *Del/+* male mice showed a deficit in object location memory compared to their wt littermate but the *Kctd13* heterozygotes were no longer defective. **c** In the NOR test, the mutant animals from the *Kctd13*^+/−^ [non-treated wt (*n* = 12), treated wt (*n* = 11), non-treated *Kctd13*^+/−^ (*n* = 14) and treated *Kctd13*^+/−^ (*n* = 10)] or the 16p11.2 *Del/+* model [non-treated wt (*n* = 17), treated wt (*n* = 9), non-treated *Del/+* (*n* = 13) and treated *Del/+* (*n* = 13)] were challenged to recognize the new object from the familiar object after a 3 h delay. *Kctd13*^+/−^ and the *Del/+* mutant mice were both impaired to recognize the new object compared to their respective wt littermates in the non-treated group. The fasudil treatment was able to restore the object recognition in the *Kctd13*^+/−^ line (*p < 0.05; **p < 0.01; ***p < 0.001)

Thus, after the behavioural characterization of the *Kctd13*^+/−^ and 16p11.2 *Del/+* mouse models, at 14 weeks of age, individuals from both genotypes were randomly assigned to two subgroups: one was an untreated control group and the other one was treated with fasudil for 4 weeks prior to further behaviour testing (Fig. 1) and then continued to be treated during the 2 weeks of testing. First, we noticed that the fasudil treatment did not change the locomotor exploration activity of the different genotypes from the *Kctd13*^+/−^ [non-treated wt (*n* = 11), treated wt (*n* = 9), non-treated *Kctd13*^+/−^ (*n* = 10) and treated *Kctd13*^+/−^ (*n* = 9)] or from the 16p11.2 *Del/+* line [non-treated wt (*n* = 20), treated wt (*n* = 18), non-treated *Del/+* (*n* = 15) and treated *Del/+* (*n* = 14)] (Fig. 3a; Additional file 1: Supplementary Fig. 3A). We revalidated that Del/+ individuals travelled more than the control wt (Two way ANOVA between groups: *F*_(1,63)_ = 28.14; *p* < 0.001; Tukey’s post hoc tests: wt vs. *Del/+:* *p* < 0.001, treated vs. non-treated: *p* = 0.791, non-treated wt vs. treated wt: *p* = 0.264, non-treated *Del/+* vs. treated *Del/+:* *p* = 0.186, non-treated wt vs. non-treated *Del/+*: *p* = 0.013 and treated wt vs. treated *Del/+:* *p* < 0.001). Interestingly, treated *Del/+* individuals experienced a less pronounced decrease in activity throughout the test (paired *t* test: *T*_0–10_ vs. *T*_20–30_ *t*_(13)_ = 2.010; *p* = 0.066). The treatment did not have an effect for any genotype on the vertical activity (Two way ANOVA between groups: *F*_(1,63)_ = 6.775; *p* = 0.01; Tukey’s post hoc tests: wt vs. *Del/+:* *p* = 0.012, treated vs. non-treated: *p* = 0.707, non-treated wt vs. treated wt: *p* = 0.743, non-treated *Del/+* vs. treated *Del/+:* *p* = 0.432, non-treated wt vs. non-treated *Del/+:* *p* = 0.2 and treated wt vs. treated *Del/+:* *p* = 0.021, Fig. 3b, Additional file 1: Supplementary Fig. 3B). As in the earlier stage, the 18-week-old *Kctd13* deficient mice were not more active in the open field than their control littermates. Interestingly, one month after the first phenotypic characterization, mutant mice from the *Kctd13*^+/−^ line [non-treated wt (*n* = 11), treated wt (*n* = 13), non-treated *Kctd13*^+/−^ (*n* = 13) and treated *Kctd13*^+/−^ (*n* = 10) littermates] performed in the NOL (Fig. 3b) but with a lower recognition index compared to wt [one sample *t* test: non treated wt (*t*_(10)_ = 3.9679; *p* = 0.0027), treated wt (*t*_(11)_ = 5.3506; *p* = 0.0002), non-treated *Kctd13*^+/−^ (*t*_(12)_ = 2.3628; *p* = 0.0359), treated *Kctd13*^+/−^ (*t*_(8)_ = 5.7266; *p* = 0.0004)]. In contrast, no improvement was observed at 18 weeks of age in heterozygous mice from the 16p11.2 *Del/+* line (non-treated wt (*n* = 13), treated wt (*n* = 15), non-treated *Del/+* (*n* = 8) and treated *Del/+* (*n* = 15) littermates) (Fig. 3b) and the fasudil treatment was not able to restore this ability in this mouse model [one sample *t* test: non-treated wt (*t*_(11)_ = 5.4167; *p* = 0.0002), treated wt (*t*_(13)_ = 4.4492; *p* = 0.0007), non-treated *Del/+* (*t*_(7)_ = 0.9837; *p* = 0.3580), treated *Del/+* (*t*_(14)_ = 2.1021; *p* = 0.0541)]*.* However, fasudil treatment significantly improved the NOR defect found in both *Kctd13*^+/−^ [non-treated wt (*n* = 12), treated wt (*n* = 11), non-treated *Kctd13*^+/−^ (*n* = 14) and treated *Kctd13*^+/−^ (*n* = 10)] and 16p11.2 *Del/+* mouse model [non-treated wt (*n* = 17), treated wt (*n* = 9), non-treated *Del/+* (*n* = 13) and treated *Del/+* (*n* = 13)] at 19 weeks of age (Fig. 3c). Here the altered recognition memory, found previously at 12 weeks, was seen again in mutants at 19 weeks and the fasudil treatment had a rescuing effect both in the *Kctd13*^+/−^ [one sample *t* test: non-treated wt (*t*_(11)_ = 2.6929; *p* = 0.0209), treated wt (*t*_(10)_ = 5.7297; *p* = 0.0002), non-treated *Kctd13*^+/−^ (*t*_(11)_ = 2.6101; *p* = 0.0243 (less than 50%), treated *Kctd13*^+/−^ (*t*_(9)_ = 3.1937; *p* = 0.0109)] and in the 16p11.2 *Del/+* mice [(One sample *t* test: non-treated wt (*t*_(16)_ = 2.3736; *p* = 0.0305), treated wt (*t*_(8)_ = 2.4481; *p* = 0.0401), non-treated *Del/+* (*t*_(12)_ = 0.3787; *p* = 0.7115), treated *Del/+* (*t*_(10)_ = 2.9168; *p* = 0.0154)]. It also highlights that at least one gene, deleted in the 16p11.2 *Del/+* model and different from *Kctd13*, contributes to the NOL phenotype in this model at this latter age.

### Molecular analyses of RHOA/ROCK signalling pathway in the ***Kctd13***^+/−^ and the 16p11.2 ***Del/+*** mouse models

**Fig. 4:**
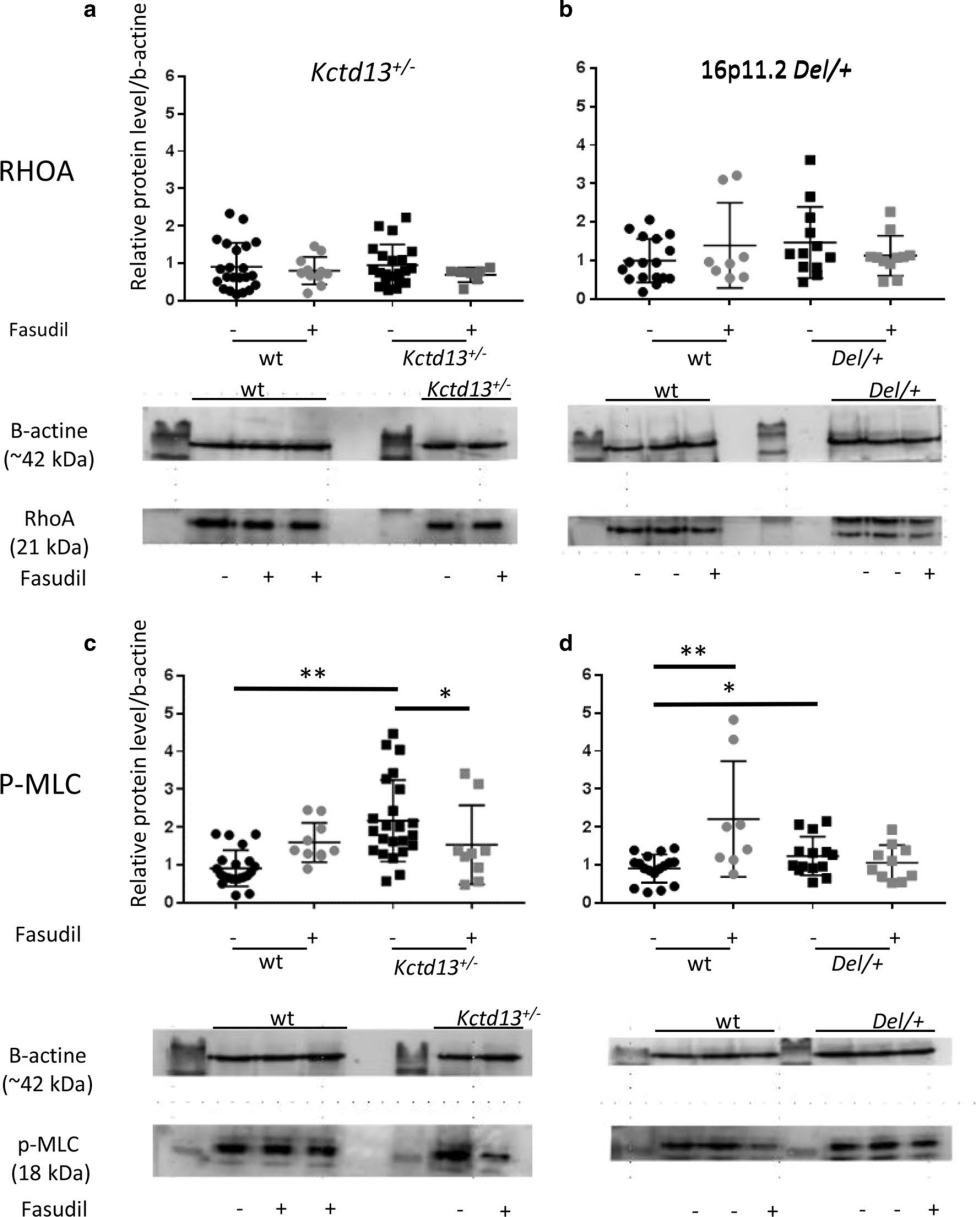
Detection of RHOA (**a**, **b**) and of the phoshorylated form of MLC (P-MLC, **c**, **d**) by western blots in heterozygous *Kctd13*^+/−^ (**a**, **c**) and the 16p11.2 *Del/+* (**b**, **d**) hippocampal lysates and their control (wt) littermate. **a** The quantification of the western blot (an example is shown below the graph) revealed no changes in RHOA protein levels in the *Kctd13*^+/−^ (A) or in the 16p11.2 *Del/+*, **b** mutant lines compared to their wt littermates. Fasudil treatment did not cause changes in RHOA protein levels in the two mutant lines [non-treated wt (*n* = 22), treated wt (*n* = 11), non-treated *Kctd13*^+/−^ (*n* = 21) and treated *Kctd13*^+/−^ (*n* = 7); and non-treated wt (*n* = 17), treated wt (*n* = 8), non-treated *Del/+* (*n* = 11) and treated *Del/+* (*n* = 11)]. However, *Kctd13* deficient mice showed an increase in the levels of phosphorylated MLC protein (**c**) and the loss of a copy of 16p11.2 region caused an increase in the levels of phosphorylated MLC protein (**d**). The treatment with fasudil reversed this alteration in *Kctd13*^+/−^ and in the *Del/+* mutant line. (*p < 0.05; **p < 0.01)

Then we checked whether the RHOA/ROCK signalling pathway was changed in both 16p11.2 deletion and *Kctd13* mouse models. To this end, we first analysed the RHOA protein levels in *Kctd13*^+/−^ and in 16p11.2 *Del/+* individuals and compared them with their respective and wild-type littermates under non-treated and treated conditions [non-treated wt (*n* = 22), treated wt (*n* = 11), non-treated *Kctd13*^+/−^ (*n* = 21) and treated *Kctd13*^+/−^ (*n* = 7); non-treated wt (*n* = 17), treated wt (*n* = 8), non-treated *Del/+* (*n* = 11) and treated *Del/+* (*n* = 11)]. Neither the deficiency of *Kctd13* nor the loss of one copy of the complete chromosomic region in 16p11.2 *Del/+* mice resulted in a change of RHOA or MLC protein levels in the hippocampal region at 18 weeks of age (Fig. 4a, b); nor did it result in a change of the MLC level in *Kctd13* and 16p11.2 Del/+ mutants versus their respective wt (Additional file 1: Supplementary Fig. 4). Nevertheless, both models showed an over-activation of the RHOA/ROCK pathway. Indeed, the Myosin Light Chain (MLC), a protein targeted by the RHOA/ROCK pathway, showed increased levels of phosphorylation in the hippocampus of non-treated *Kctd13*^+/−^ and 16p11.2 *Del/+* compared to their respective wt littermates [*Kctd13*^+/−^ (non-treated wt (*n* = 21), treated wt (*n* = 10), non-treated *Kctd13*^+/−^ (*n* = 23) and treated *Kctd13*^+/−^ (*n* = 9) and Del/+ (non-treated wt (*n* = 17), treated wt(*n* = 8), non-treated *Del/+* (*n* = 14) and treated *Del/+* (*n* = 10)] mice, supporting the idea that this pathway could be associated with the cognitive phenotype observed (Fig. 4c, d; Additional file 1: Table 4). Therefore, we verified whether the therapeutic effect of fasudil acted through the inhibition of this RHOA/ROCK signalling pathway. Interestingly, the fasudil treatment reduced MLC phosphorylation levels in *Kctd13* mutant individuals (Kruskal–Wallis one-way analysis of variance between groups: *H*_(3)_ = 21.731; *p* < 0,001; Mann–Whitney test: non treated wt versus non treated *Kctd13*^+/−^: *p* = 0.009; non treated wt versus treated *Kctd13*^+/−^: *p* = 0.702; non treated *Kctd13*^+/−^ versus treated *Kctd13*^+/−^: *p* = 0.049). As for the *Kctd13*^+/−^ model, we found that in 16p11.2 deficient mice fasudil restored normal MLC phosphorylation in treated mutant mice but, surprisingly, induced increased MLC phosphorylation in wt mice (Kruskal–Wallis one-way analysis of variance between groups: *H*_(3)_ = 8.457; *p* = 0.037; Mann–Whitney test: non treated wt versus treated wt: *p* = 0.008; *t* test: non treated wt versus non treated *Del/+:* *p* = 0.047, non -reated wt versus treated *Del/+*: *p* = 0.364).

## Discussion

Here, we described the phenotypes of another *Kctd13*^+/−^ mouse mutant line that replicates some of the defects seen in young mouse models carrying the 16p11.2 BP4-BP5 deletion, and we explored a treatment aimed at reducing the activity of the RHOA/ROCK pathway on cognition. With the new *Kctd13*^+/−^ mouse line, we were able to detect changes in the NOL and NOR, but no effect on the exploration activity compared to the 16p11.2 *Del/+* mouse model. These results are in agreement with a recent study highlighting the role of KCTD13 in the 16p11.2 deletion syndrome [24]. The loss of one copy of *Kctd13* gene did not cause alterations on the exploration or vertical activity of mice in the open field test. However, the hemi-deletion of the entire 16p11.2 region induced hyperactivity in these animals. This observation leads us to propose that *Kctd13* genetic dosage is not involved in the increased exploration activity associated with 16p11.2 deletion. Accordingly, chronic fasudil administration did not attenuate the hyper locomotion affecting 16p11.2 *Del/+* mice. This finding suggests that there could be other genes of the region involved in this phenotype. Consequently, the treatment with an inhibitor of the RHOA/ROCK pathway did not produce any effect on this phenotype.

When we analysed the NOL in *Kctd13* mutant mice, we found an impairment in the novelty detection in the NOL test at 12 weeks of age. However, at 18 weeks when we repeated the test, the non-treated mutant mice showed an improved recognition index. This observation may show that either there is a maturation deficit that is recovered in older mice, perhaps through compensatory effects, or there could be an effect in the repetition of the test. We favour the first hypothesis as the NOR/NOL phenotypes in this line were found at both ages in the 16p11.2 deletion. Indeed, we found an NOL deficit for the 16p11.2 *Del/+* mice at 12 weeks, and which was still present at 18 weeks, not corrected by chronic administration of fasudil for 5 weeks. Thus, further research will be necessary to analyse how this *Kctd13*-induced NOL phenotype is rescued in 18-week-old non-treated mice. This finding confirms that the NOL-phenotype associated with the 16p11.2 rearrangement is not completely dependent on *Kctd13* dosage. Several genes can contribute to the NOL phenotype on top of the *Kctd13* gene. Indeed, loss of the serine/threonine Tao kinase 2 leads to brain and behavioural abnormalities with altered dendritic growth and synaptic connectivity through RHOA signalling [30].

The object recognition memory deficit is one of the most robust and reproducible phenotypes associated with 16p11.2 *Del/+* mice [20]. In agreement with previous research, our *Kctd13*^+/−^ mouse model has deficits in novelty detection. Furthermore, our study showed that fasudil treatment significantly rescued this impairment in mutant mice. Thus, the loss of KCTD13 is one of the main drivers of the object recognition phenotype associated with 16p11.2 deletion. In addition, the therapeutic effects of fasudil on *Kctd13* deficient and 16p11.2 deletion mice highlight the role of the RHOA/ROCK signalling pathway as the main mechanism responsible for this phenotype.

Interestingly, individuals lacking the *Kctd13* gene showed no change in expression levels of the RHOA protein and fasudil treatment did not modify RHOA protein expression in mutant and control mice. Likewise, the carriers of the 16p11.2 hemi-deletion did not display changes of expression for this protein. Although we were not able to detect a change in the RHOA protein level, *Kctd13* mutants and 16p11.2 deficient mice had increased phosphorylated-MLC levels. This observation confirms that RHOA/ROCK pathway outcome is over-activated due to a copy loss of *Kctd13* in *Kctd13* and 16p11.2 mutants. Furthermore, our study showed that the therapeutic effect of fasudil on the recognition memory phenotype associated with 16p11.2 deletion was somehow due to the normalizing action of the drug in both mouse models.

At this point, we can highlight the clinical relevance of the treatment because of its potential as a cognitive enhancer in humans with memory and learning dysfunction related to neurodevelopmental disorders. However, more work is necessary to understand the precise molecular mechanism affected by the loss of the *Kctd13* gene causing the over-activation of the RHOA/ROCK pathway.

We consider that further biochemical studies of the RHOA/ROCK pathway and the KCTD13-CULLIN3 complex are needed to understand the mechanistic role of the pathway in the syndromes associated with 16p11.2 rearrangements. In addition, our results do not ignore that other genes in the region may act at other levels of the RHOA pathway or at different times in development [30]. Probably, several genes play a similar role, or the expression of a gene may regulate the expression of another gene in the region, considering the high genotypic density of the 16p11.2 interval and the variability of neurological-associated phenotypes [31, 32].

## Limitations

In this study we found some similarities between *Kctd13* and 16p11.2 deletion models, not only in terms of phenotypes but also in terms of rescue for the novel object location and object recognition paradigms. This analysis is limited as it was done in a pure genetic background where some of the Del/+ mutants were not viable. Thus, this study was limited to a subset of viable mutant animals carrying the 16p11.2 deletion. We could have improved this because these models can display normal post-natal viability on an F1 genetic background with an additional defect in social behaviour [20]. Nevertheless, we will have to carry out a similar study with the *Kctd13* null allele. Overall, this study was performed on a mouse model that replicates only a partial set of features found in humans with 16p11.2 deletion, thus our conclusion only provides a partial response for the human condition.

## Conclusion

Here we showed that *Kctd13* haploinsufficiency phenocopied the object location and recognition memory deficits seen in the 16p11.2 deletion model but did not change exploration activity in the open field. Treatment of adult mice with fasudil, an inhibitor of RHOA, was able to restore novel object location and recognition memories. Furthermore, fasudil treatment was able to restore the RHOA/ROCK signalling pathway to an almost normal level of phosphorylation of the Myosin Light Chain in the brain.

## Supplementary Information

**Additional file 1.** Supplementary data.

## Data Availability

All the mouse models are available either through the Infrafrontier research infrastructure (16p11.2 deletion, www.infrafrontier.eu; EM:06,133) or the international mouse phenotyping consortium (Kctd13 mutant, www.mousephenotype.org). The datasets analysed during the current study are available from the corresponding author on reasonable request.

## Abbreviations

Del: Deletion
MLC: Myosin light chain
NOL: Novel object location
NOR: Novel object recognition
OF: Open field
RI: Recognition index
wt: Wild-type
