# Supplementary material for: Targeting the RHOA pathway improves learning and memory in adult Kctd13 and 16p11.2 deletion mouse models
Source: Mol Autism. 2021 Jan 13;12:1. doi: 10.1186/s13229-020-00405-7 (PMC7805198; doi:10.1186/s13229-020-00405-7)
Supplement: Supplementary file 1 — Additional file 1. Supplementary data. [file 13229_2020_405_MOESM1_ESM.docx]

**SUPPLEMENTARY MATERIALS**

Targeting the RHOA pathway improves learning and memory in adult *Kctd13* and 16p11.2 deletion mouse models.

**Authors**

Sandra MARTIN LORENZO^1^, Valérie NALESSO**^1^**, Claire CHEVALIER**^1^**, Marie Christine BIRLING^2^, and Yann HERAULT**^1,2,*^**.

**Affiliations**

^1^ Université de Strasbourg, CNRS, INSERM, Institut de Génétique Biologie Moléculaire et Cellulaire, IGBMC - UMR 7104 - Inserm U1258, 1 rue Laurent Fries, 67404 ILLKIRCH cedex

^2^ Université de Strasbourg, CNRS, INSERM, CELPHEDIA-PHENOMIN-ICS, Institut Clinique de la Souris, 1 rue Laurent Fries, 67404 ILLKIRCH cedex

*Corresponding author: Yann HERAULT, herault@igbmc.fr

**Keywords:** Copy number variation, neurodevelopment, intellectual disability, autism spectrum disorders, KCTD13, mouse model, recognition memory.

**Running title:** Therapeutic effects of fasudil in *Kctd13* and 16p11.2 deficient mouse models.

Supplementary TABLES

|  | | Pre treatment | | Post treatment | | | | |
| --- | --- | --- | --- | --- | --- | --- | --- | --- |
|  | | **wt** | ***Kctd13^+/-^*** | **wt** | | ***Kctd13^+/-^*** | | |
| Test | **Parameter** |  |  | Non treated | **Treated** | **Non treated** | | **Treated** |
| Open Field | Total distance (m) | 29 % | | 11 % | | | | |
|  | Rears (count) | 60 % | | <10 % | | | | |
| Novel Object Location Recognition  5 min delay | Recognition Index (%) | 100 % | 99 % | 100 % | 100 % | 100 % | 100 % | |
| Novel Object Recognition  3 hours delay | Recognition Index (%) | 100 % | 100% | 100 % | 100 % | 96 % | 100 % | |

|  | | Pre treatment | | Post treatment | | | |
| --- | --- | --- | --- | --- | --- | --- | --- |
|  | | **wt** | **16p11.2**  ***Del/+*** | **wt** | | **16p11.2**  ***Del/+*** | |
| Test | **Parameter** |  |  | Non treated | Treated | Non treated | Treated |
| Open Field | Total distance (m) | 100 % | | 100 % | | | |
|  | Rears (count) | 100 % | | 61 % | | | |
| Novel Object Location Recognition  5 min delay | Recognition Index (%) | 100 % | 100 % | 100 % | 100 % | 80 % | 100 % |
| Novel Object Recognition  3 hours delay | Recognition Index (%) | 100 % | 95% | 100 % | 100 % | 100% | 100 % |

**Sup. Table 1: A posteriori power calculation for the variable used in the behavioural analysis.** The power calculation was based on the data collected in the behavioural analysis and the number of samples used. We fixed the alpha error to 0.02 and we calculated the effect size.

|  | | Pre treatment | | Post treatment | | | |
| --- | --- | --- | --- | --- | --- | --- | --- |
|  | | **wt** | ***Kctd13^+/-^*** | **wt** | | ***Kctd13^+/-^*** | |
| Test | **Parameter** |  |  | **Non treated** | **Treated** | **Non treated** | **Treated** |
| Open Field | Total distance (m) | 94 ± 4 | 92 ± 5 | 81 ± 5 | 91 ± 6 | 82 ± 5 | 88 ± 8 |
|  | Distance t 0-10 (m) | 40 ± 1 | 38 ± 2 | 34 ± 2 | 35 ± 3 | 33 ± 2 | 34 ± 3 |
|  | Distance t 10-20 (m) | 30 ± 1 | 30 ± 2 | 26 ± 1 | 29 ± 2 | 27 ± 2 | 27 ± 3 |
|  | Distance t 20-30 (m) | 25 ± 2 | 27 ± 2 | 21 ± 2 | 26 ± 2 | 24 ± 2 | 25 ± 3 |
|  | Rears (count) | 244 ± 13 | 234 ± 15 | 149 ± 22 | 155 ± 14 | 148 ± 21 | 157 ± 20 |
| Novel Object Location Recognition  5 min delay | S1 object exploration (s) | 9 ± 1 | 10 ± 1 | 7 ± 1 | 6 ± 1 | 8 ± 1 | 7 ± 1 |
|  | S2 non-displaced object exploration (s) | 3 ± 0,4 | 6 ± 1* | 2 ± 0,4 | 3 ± 0,4 | 4 ± 1 | 3 ± 0,4 |
|  | S2 displaced object (s) | 6 ± 1 | 6 ± 1 | 4 ± 0,4 | 6 ± 1 | 5 ± 1 | 6 ± 1 |
|  | Recognition Index (%) | 65 ± 3^§§§^ | 52 ± 2*** | 68 ± 5^§§^ | 66 ± 3^§§§^ | 55 ± 2^§^ | 64 ± 2^§§§^ |
| Novel Object Recognition  3 hours delay | S1 object A exploration (s) | 18 ± 2 | 19 ± 3 | 13 ± 2 | 30 ± 5* | 19 ± 4 | 16 ± 3 |
|  | S2 object A exploration (s) | 5 ± 1 | 7 ± 1 | 6 ± 1 | 5 ± 1 | 6 ± 1 | 4 ± 1 |
|  | S2 object B exploration (s) | 7 ± 1 | 5 ± 1 | 10 ± 2 | 8 ± 2 | 7 ± 2 | 8 ± 2 |
|  | Recognition Index (%) | 57 ± 2^§§^ | 50 ± 3 | 62 ± 4^§^ | 61 ± 2^§§§^ | 46 ± 4 | 66 ± 5^§^ |

**Sup. Table 2.** Behavioural characterization of the *Kctd13^+/-^* mouse model before and after fasudil chronic treatment. In the open field test, no change of horizontal (total distance) or vertical activity (rears) was detected because the inactivation of the gene pre or post treatment. Recognition memory was analysed through the novel object location and object recognition test. *Kctd13^+/-^* mice showed a deficit for displaced object discrimination and novel object recognition. Mutant males presented no recognition indexes significantly higher than the level of chance 50%. Data are mean ± SEM. Test de Student, * *p* < 0.05, ****p* < 0.001. One Sample t-test, ^§§^*p* < 0.01, ^§§§^*p* < 0.001 compared with the chance level (50%). Each genotype was divided into 2 groups, non-treated and treated mice. Non-treated *Kctd13^+/-^* mice showed 4 weeks later improvements for novel object location recognition index. In the novel object recognition test, non-treated *Kctd13^+/-^* mice showed 4 weeks later a deficit for novel object recognition index while treated *Kctd13^+/-^* mice recovered the object recognition memory. Data are mean ± SEM. Tukey’s test, * *p* < 0.05. One Sample t-test, ^§^*p* < 0.05, ^§§^*p* < 0.01, ^§§§^*p* < 0.001 compared with the chance level (50%).

|  | | Pre treatment | | Post treatment | | | |
| --- | --- | --- | --- | --- | --- | --- | --- |
|  | | **wt** | **16p11.2 *Del/+*** | **wt** | | **16p11.2**  ***Del/+*** | |
| Test | **Parameter** |  |  | **Non treated** | **Treated** | **Non treated** | **Treated** |
| Open Field | Total distance (m) | 91 ± 2 | 105 ± 3*** | 76 ± 3 | 71 ± 4 | 91 ± 4 | 99 ± 5*** |
|  | Distance t 0-10 (m) | 35 ± 1 | 42 ± 1 | 30 ± 2 | 29 ± 2 | 34 ± 2 | 35 ± 2 |
|  | Distance t 10-20 (m) | 29 ± 1 | 33 ± 1 | 26 ± 1 | 23 ± 1 | 30 ± 1 | 34 ± 2 |
|  | Distance t 20-30 (m) | 27 ± 1 | 29 ± 1 | 21 ± 1 | 18 ± 1 | 26 ± 2 | 30 ± 2 |
|  | Rears (count) | 217 ± 13 | 253 ± 17 | 129 ± 13 | 123 ± 11 | 154 ± 15 | 171 ± 17 |
| Novel Object Location Recognition  5 min delay | S1 object exploration (s) | 10 ± 1 | 16 ± 2** | 7 ± 1 | 9 ± 1 | 12 ± 2 | 10 ± 1 |
|  | S2 non-displaced object exploration (s) | 4 ± 0,4 | 6 ± 1** | 3 ± 0,4 | 2 ± 0,4 | 5 ± 1** | 4 ± 1 |
|  | S2 displaced object (s) | 5 ± 1 | 7 ± 1 | 6 ± 1 | 6 ± 1 | 4 ± 1 | 6 ± 1 |
|  | Recognition Index (%) | 58 ± 2^§§^ | 54 ± 2 | 68 ± 3^§§§^ | 69 ± 4^§§§^ | 45 ± 5** | 58 ± 4 |
| Novel Object Recognition  3 hours delay | S1 object A exploration (s) | 16 ± 2 | 20 ± 2 | 15 ± 2 | 11 ± 2 | 30 ± 5* | 24 ± 5 |
|  | S2 object A exploration (s) | 5 ± 1 | 9 ± 1** | 4 ± 1 | 4 ± 1 | 10 ± 2* | 6 ± 1 |
|  | S2 object B exploration (s) | 6 ± 1 | 8 ± 1 | 6 ± 1 | 6 ± 1 | 9 ± 2 | 9 ± 1 |
|  | Recognition Index (%) | 58 ± 3^§^ | 52 ± 3 | 59 ± 4^§^ | 59 ± 4^§^ | 49 ± 4 | 59 ± 3^§^ |

**Sup. Table 3.** Behavioural characterization of the 16p11.2 *Del/+* mouse model before and after fasudil chronic treatment. In the pre-treatment open field test, the mutant mice showed increased horizontal activity during the 30 minutes of test (total distance) and during the first-time intervals. Recognition memory was analysed in our mouse model through the novel object location and object recognition test. In general, *Del/+* mice spent more time exploring the objects during session S1. This higher exploration is due to the increased exploration activity associated with the loss of one copy of the 16p11.2 region. Del/+ mice showed a deficit for displaced object discrimination and novel object recognition. Data are mean ± SEM. Test de Student, ***p* < 0.01, ****p* < 0.001. One Sample t-test, ^§^*p* < 0.05, ^§§^*p* < 0.01 compared with the chance level (50%). Our animals were divided into 4 different groups depending on their genotype and treatment. Post-treatment analyses showed for the open field test a higher difference in exploration activity between the treated mutant group and the control group. In the novel object location test, non-treated *Del/+* animals showed a deficit for displaced object recognition. Treatment in mutant mice increased recognition index but it was not significantly higher than the level of chance (50%). Data are mean ± SEM. Tukey’s test, * *p* < 0.05, ***p* < 0.01, ****p* < 0.001. One Sample t-test, ^§§§^*p* < 0.001 compared with the chance level (50%). In novel object recognition test, non-treated mutant mice showed 4 weeks later a deficit for novel object recognition index while fasudil treatment rescued the object recognition memory in mutant mice. Data are mean ± SEM. Mann-Witney *U* test, * *p* < 0.05. One Sample t-test, ^§^*p* < 0.05 compared with the chance level (50%).

|  | wt | | | | *Kctd13^+/-^* | |  |
| --- | --- | --- | --- | --- | --- | --- | --- |
| Protein | | **Non treated** | **Treated** | **Non treated** | | **Treated** | |
| RHOA | | 0.91 ± 0.14 | 0.81 ± 0.11 | 0.96 ± 0.12 | | 0.7 ± 0.07 | |
| p-MLC | | 0.92 ± 0.1 | 1.6 ± 0.17 | 2.17 ± 0.22** | | 1.54 ± 0.35 | |
|  | | **wt** | | **16p11.2 *Del/+*** | | | |
| Protein | | **Non treated** | **Treated** | **Non treated** | | **Treated** | |
| RHOA | | 1 ± 0.14 | 1.4 ± 0.4 | 1.47 ± 0.27 | | 1.13 ± 0.16 | |
| p-MLC | | 1 ± 0.1 | 2.42 ± 0.59** | 1.36 ± 0.15* | | 1.16 ± 0.16 | |

**Sup. Table 4.** RHOA protein and MLC phosphorylation levels analysed by western blot from hippocampal regions of *Kctd13^+/-^* mouse model treated or non-treated with fasudil Chronic treatment. The intensity of each interest protein bands normalized with the intensity of the corresponding control protein band was again normalized with the mean of all samples of the untreated control individuals. Non-treated *Kctd13^+/-^* hippocampus presented increased phosphorylation levels of MLC protein while mutant treated with fasudil didn’t present significant increased phosphorylation levels of protein. In the case of the mouse model for the deletion of the 16p11.2 region, we also found an increased phosphorylation levels of MLC. These levels were normalized with the treatment. Mann-Witney *U* test, * *p* < 0.05, ** *p* < 0.01.

Supplementary FIGUREs


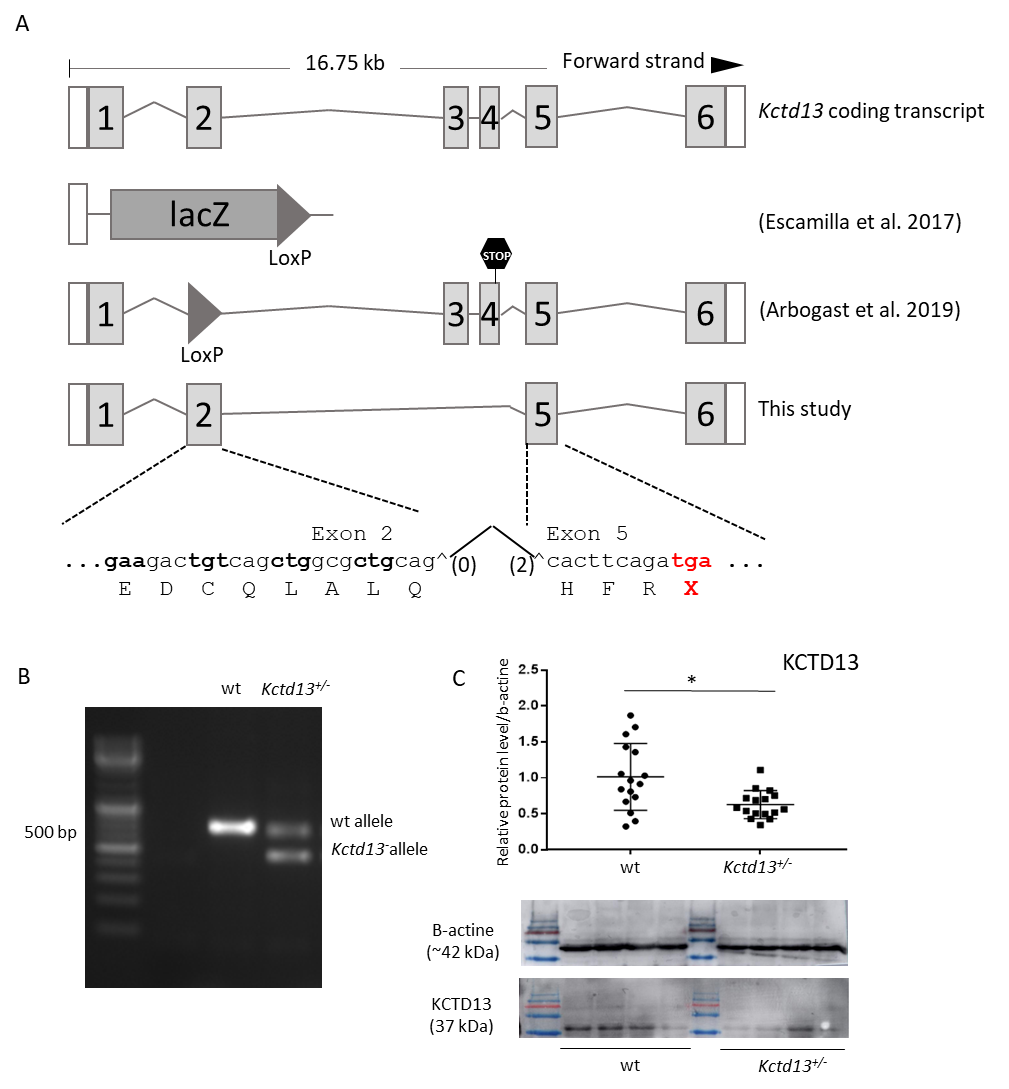


**Sup. Fig.1. A new CrispR/Cas9 engineered *Kcdt13* loss-of-function allele** (A) Representation of the *Kctd13* alleles used in previous publications and in the present study. The allele generated by Escamilla et al. [1] correspond to a deletion of the whole locus and the insertion of a lacZ gene reporter. In Arbogast et al.[2], the deletion of exon 2 leads to a frameshift in exon 4 while the removal of exons 3 and 4 in our allele leads to a change from frame 0 in exon2 to frame +2 in exon 5 and a premature stop codon. (B) Molecular validation by PCR showed products specific for the wt and *Kctd13^+/-^* alleles of 668-bp and 429-bp long, respectively. (C) Western blot confirmed the decreased level of KCTD13 protein in mutant mice hippocampus region (wt (n=16) and *Kctd13^+/-^* (n=16)). (Mann-Whitney U Statistic = 64; *p* = 0,017). (* *p* < 0.05).

**
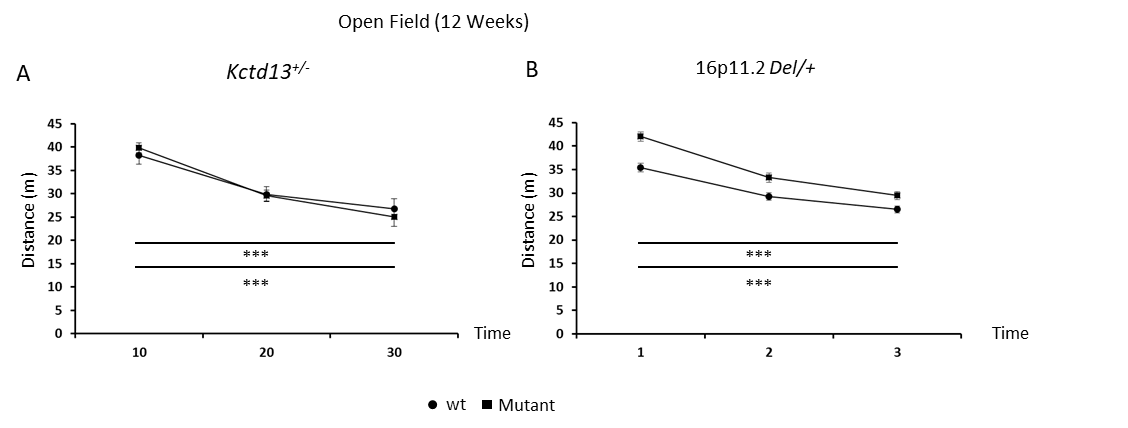
**

**Sup. Fig.2. Distance traveled in the open field by the *Kctd13^+/-^* (A) and *Del/+* (B) animals showed a normal habituation to the new environment with a significant decreased of the arena exploration during the test.** Paired t test: wt: T_0-10_  vs. T_20-30_ t_(19)_ = 7,507; *p* < 0,001; *Kctd13^+/-^* : T_0-10_  vs. T_20-30_ t_(16)_ = 4,453; *p* < 0,001. Paired t test: wt: T_0-10_  vs. T_20-30_ t_(37)_ = 10,834; *p* < 0,001; *Del/+*: T_0-10_  vs. T_20-30_ t_(31)_ = 14,319; *p* < 0,001.


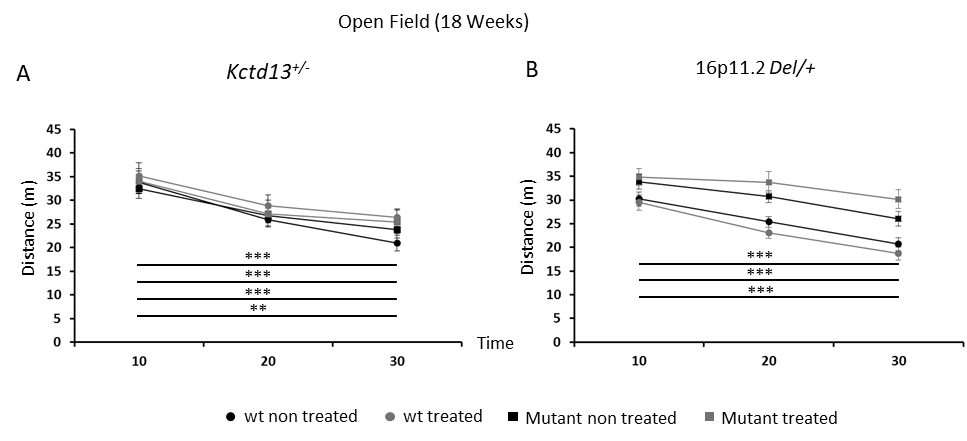


**Sup. Fig.3. Habituation to a new environment is not altered in the *Kctd13* and 16p11.2 deletion models at 18 weeks of ages or by the treatment.** The *Kctd13^+/-^* (A) individuals showed a normal habituation to the new environment with a significant decreased of the arena exploration during the test, which was not affected by the fasudil treatment. Paired t test: non treated wt: T_0-10_  vs. T_20-30_ t_(10)_ = 5,970; *p* < 0,001; treated wt: T_0-10_  vs. T_20-30_ t_(8)_ = 5,117; *p* < 0,001; non treated *Kctd13^+/-^* : T_0-10_  vs. T_20-30_ t_(10)_ = 5,543; *p* < 0,001; treated *Kctd13^+/-^* : T_0-10_  vs. T_20-30_ t_(9)_ = 3,290; *p* = 0.009. Whereas for the *Del/+* mice (B) fasudil affected the habituation to the environment. Paired t test: non treated wt: T_0-10_  vs. T_20-30_ t_(19)_ = 6,939; *p* < 0,001; treated wt: T_0-10_  vs. T_20-30_ t_(18)_ = 9,753; *p* < 0,001; non treated *Del/+*: T_0-10_  vs. T_20-30_ t_(14)_ = 6,516; *p* < 0,001; treated *Del/+*: T_0-10_  vs. T_20-30_ t_(13)_ = 2,010; *p* = 0.066.


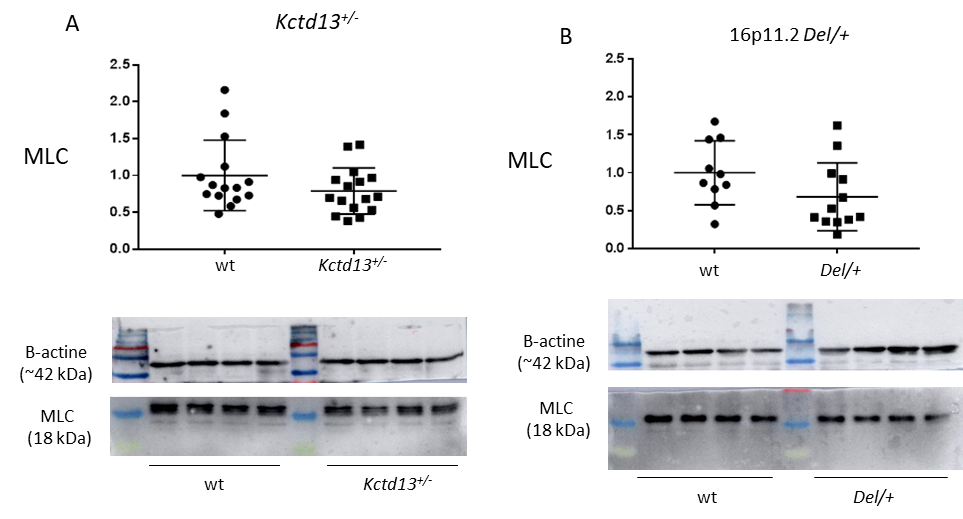


**Sup. Fig.4. Western blot against MLC showed that (A) *Kctd13* deficiency or (B) 16p11.2 Del/+ models does not affect total level protein in mice’s hippocampus region.** A: wt (n=15) and *Kctd13^+/-^* (n=16)). (Mann-Whitney U Statistic = 86; *p* = 0,185). B: wt (n=10) and *Del/+* (n=12)). (Student t test: t_(20)_ = 1,701; *p* = 0,104).

REFERENCES

1. Escamilla CO, Filonova I, Walker AK, Xuan ZX, Holehonnur R, Espinosa F, Liu S, Thyme SB, López-García IA, Mendoza DB *et al*: **Kctd13 deletion reduces synaptic transmission via increased RhoA**. *Nature* 2017, **551**(7679):227-231.

2. Arbogast T, Ouagazzal AM, Chevalier C, Kopanitsa M, Afinowi N, Migliavacca E, Cowling BS, Birling MC, Champy MF, Reymond A *et al*: **Reciprocal Effects on Neurocognitive and Metabolic Phenotypes in Mouse Models of 16p11.2 Deletion and Duplication Syndromes**. *PLoS Genet* 2016, **12**(2):e1005709.
